# Immunomodulation of HDAC Inhibitor Entinostat Potentiates the Anticancer Effects of Radiation and PD-1 Blockade in the Murine Lewis Lung Carcinoma Model

**DOI:** 10.3390/ijms232415539

**Published:** 2022-12-08

**Authors:** Yeeun Kim, Kyunghee Park, Yeon Jeong Kim, Sung-Won Shin, Yeon Joo Kim, Changhoon Choi, Jae Myoung Noh

**Affiliations:** 1 Department of Radiation Oncology, Samsung Medical Center, Seoul 06351, Republic of Korea; 2 Samsung Genome Institute, Samsung Medical Center, Seoul 06351, Republic of Korea; 3 Department of Radiation Oncology, Sungkyunkwan University School of Medicine, Seoul 06351, Republic of Korea

**Keywords:** entinostat, radiation, anti-PD-1, antitumor immunity, lung cancer

## Abstract

Although the combination of radiotherapy and immunotherapy has proven to be effective in lung cancer treatment, it may not be sufficient to fully activate the antitumor immune response. Here, we investigated whether entinostat, a histone deacetylase inhibitor, could improve the efficacy of radiotherapy and anti-PD-1 in a murine syngeneic LL/2 tumor model. A total of 12 Gy of X-rays administered in two fractions significantly delayed tumor growth in mice, which was further enhanced by oral entinostat administration. Flow cytometry-aided immune cell profiling revealed that entinostat increased radiation-induced infiltration of myeloid-derived suppressor cells and CD8^+^ T cells with decreased regulatory T-cells (Tregs). Transcriptomics-based immune phenotype prediction showed that entinostat potentiated radiation-activated pathways, such as JAK/STAT3/interferon-gamma (IFN-γ) and PD-1/PD-L1 signaling. Entinostat augmented the antitumor efficacy of radiation and anti-PD-1, which may be related to an increase in IFN-γ-producing CD8^+^ T-cells with a decrease in Treg cells. Comparative transcriptomic profiling predicted that entinostat increased the number of dendritic cells, B cells, and T cells in tumors treated with radiation and anti-PD-1 by inducing MHC-II genes. In conclusion, our findings provided insights into how entinostat improves the efficacy of ionizing radiation plus anti-PD-1 therapy and offered clues for developing new strategies for clinical trials.

## 1. Introduction

Lung cancer is the second most common cancer worldwide and its incidence continues to increase. Radiation therapy (RT) is a major component of the multimodal treatment for patients with solid cancers, including lung cancer. RT is a double-edged sword that induces an antitumor immune response and promotes immune suppression [1]. For example, RT encourages the recruitment of cytotoxic killer T cells and immunosuppressive cells, such as myeloid-derived suppressive cells (MDSCs) and regulatory T cells (Tregs). Immune checkpoint inhibitors (ICIs) designed to block the programmed cell death-1 (PD-1) and programmed cell death-ligand 1 (PD-L1) pathways have revolutionized lung cancer treatment. Combined with the PD-1 signaling blockade, ICIs reinvigorate RT-induced immune suppression, resulting in a better outcome. The phase 3 PACIFIC study showed that concurrent chemoradiation followed by consolidation with durvalumab (anti-PD-L1) resulted in longer progression-free survival and a higher overall survival rate than placebo [2,3]. Nevertheless, the combination of RT and ICIs may not be sufficient to fully activate the antitumor immune response, and other combination strategies need to be considered to optimize efficacy.

Histone deacetylases (HDACs) can remodel chromatin structures via histone modification and modulate the expression of malignancy-related genes, making them attractive targets for cancer treatment. Preclinical studies have shown that HDAC inhibitors (HDACis) exhibit antitumor activity against non-small lung cancer (NSCLC) cell lines by inducing apoptotic pathways [4,5]. In clinical trials, HDACis, combined with conventional chemotherapeutic agents, such as paclitaxel and erlotinib, have been tested in patients with NSCLC [6,7]. Several HDACis, including vorinostat and abexinostat, showed radiosensitizing activity in NSCLC cells [8,9]. Other HDACis, such as valproic acid and panobinostat, synergize with particle beams, such as protons, to potentiate hepatocellular carcinoma cell death [10,11]. Besides cancer cells, HDACis are also known to rewire the signaling of many other cells, including immune cells, in the tumor microenvironment (TME).

Recently, HDACi immunomodulation has gained great interest in immuno-oncology. In the cancer-immunity cycle, HDAC inhibition enhances antigen presentation by increasing major histocompatibility complex (MHC) class I and II in Merkel cell carcinoma [12], bladder cancer [13], and NSCLC [14], resulting in the promotion of cytotoxic CD8+ T-cell-mediated tumor cell killing. HDACis upregulate PD-L1, an immune checkpoint protein, which augments PD-1 blockade efficacy in syngeneic B16F10 and 4T1 mouse models [15,16,17]. HDACis modulate the expression of chemokines and cytokines, thereby affecting immunosuppressive cells, including Tregs and MDSCs, in various cancer models [14,16,18,19,20,21]. Many ongoing clinical trials are investigating the potential benefits of combining HDACis with ICIs in solid cancer treatment [22].

Despite many efforts to test HDACis in combination with either radiation or ICIs, little is known about the therapeutic effect of triple combination therapy. This study aimed to investigate the potential of entinostat, a class I HDAC-selective inhibitor, on RT or a combination of RT and anti-PD-1 using a syngeneic murine Lewis lung carcinoma LL/2 model. Previous studies have demonstrated that entinostat synergizes with ICIs in murine lung, kidney, breast, and pancreatic cancer mouse models [19,21]. Our findings provided evidence that entinostat can boost the immune-RT combination. Immune cell profiling and transcriptomic analysis revealed that entinostat enhanced the antitumor effect of RT or RT plus anti-PD-1 by increasing effector T-cell infiltration and MHC-II expression.

## 2. Results

### 2.1. Entinostat Enhances Radiation-Induced Tumor Growth Delay in a Lewis Lung Cancer Model

**Figure 1:**
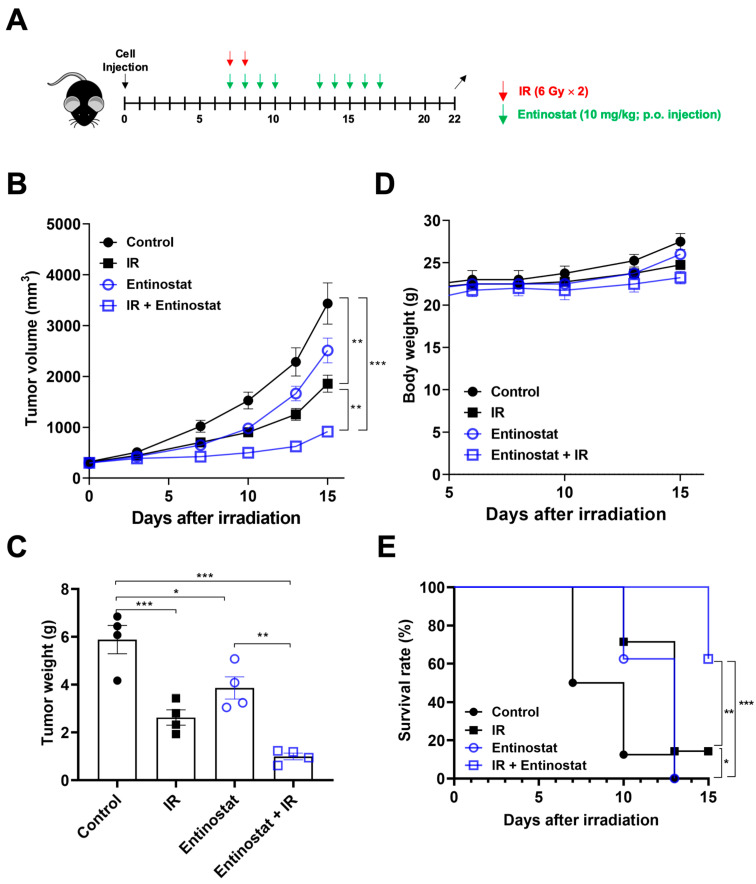
Entinostat increases radiation-induced tumor growth delay in a Lewis lung cancer model. (**A**) Scheme for radiation and entinostat treatments. (**B**) Tumor growth curves showing reduced tumor growth in LL/2 tumor-bearing C57/BL6 mice treated with entinostat and/or IR. (**C**) Comparison of tumor weight at day 15 after irradiation. (**D**) Body weight changes of LL/2 tumor-bearing mice after treatments. (**E**) Survival curves of LL/2 tumor-bearing mice after entinostat and/or IR treatment. Data are mean ± S.D. (*n* = 8). * *p* < 0.05; ** *p* < 0.01; *** *p* < 0.001. Mice were categorized as dead when the tumor volume reached 2000 mm^3^.

To determine whether entinostat enhances radiation efficacy through immune modulation, we used a syngeneic Lewis LL/2 lung tumor model. Based on our previous result that three 6 Gy fractions (total of 18 Gy) may be strong enough to suppress the growth of LL/2 tumors [23], we chose two 6 Gy fractions for the present study. LL/2 tumors were allowed to grow after being injected into the right hind legs of C57BL/6 mice. Then, they were irradiated with X-rays 7 d after cell injection (Figure 1A). Entinostat was orally administered every day for 2 weeks starting on the same day as irradiation. Both entinostat and ionizing radiation (IR) alone decreased tumor volume and weight on day 15 after irradiation (Figure 1B,C). Significant tumor growth inhibition was observed in the combination group compared to that in the single treatment group (Figure 1B,C). The combined effect of IR and entinostat was additive, based on tumor growth reduction. No weight loss was observed during treatment (Figure 1D). Survival analysis of LL/2-bearing mice showed that the combined treatment prolonged survival compared to the single treatment (Figure 1E).

### 2.2. Entinostat Reshapes Immune Response in the Tumors That Receive Radiotherapy

**Figure 2:**
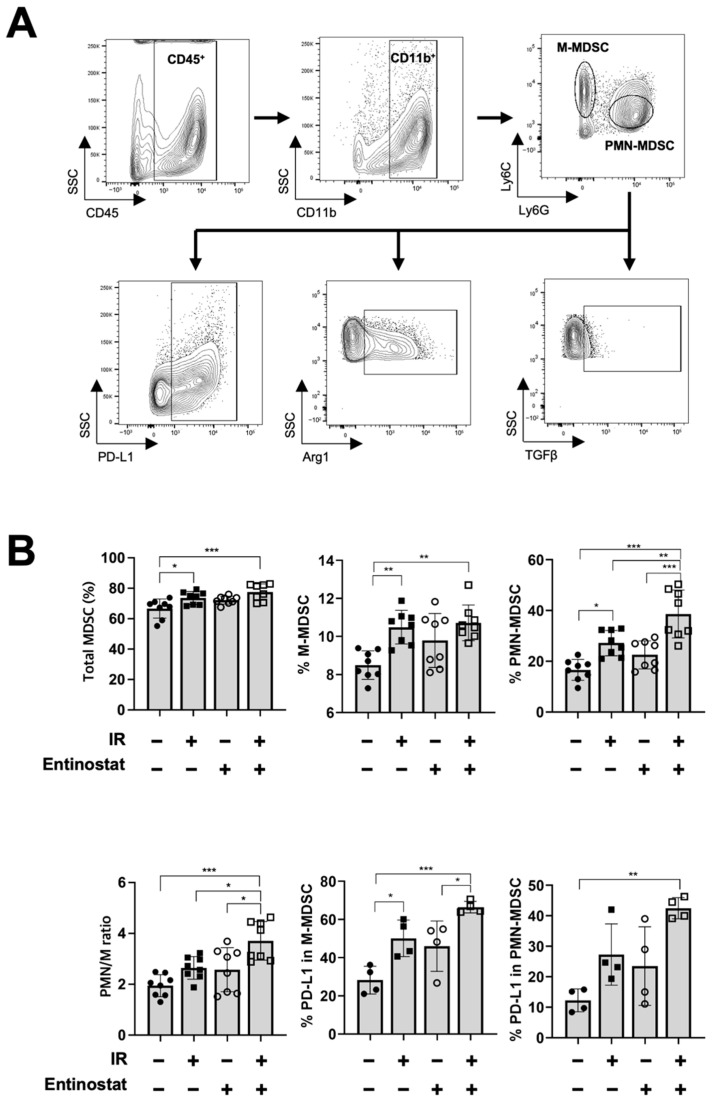
Entinostat modulates radiation-induced MDSC infiltration in tumors. (**A**) Gating strategy for the evaluation of the myeloid-derived suppressor cells (MDSC) population using flow cytometry. Representative density plots are shown. (**B**) Flow cytometric analysis of MDSC populations. Radiation increased the infiltration of total MDSCs in tumors. Entinostat further enhanced PMN-MDSC but not M-MDSC. PD-L1 expression on MDSCs was further increased by IR and entinostat combination. Data are mean ± S.D. (*n* ≥ 4). * *p* < 0.05; ** *p* < 0.01; *** *p* < 0.001.

Next, we performed flow cytometry-based immune profiling to determine how entinostat modulates antitumor immunity. Since entinostat is known to decrease MDSC populations within tumors [19,21], we determined the abundance and activity of MDSCs infiltrating tumors harvested 15 d after irradiation. Two major subpopulations of MDSCs, monocytic MDSCs (M-MDSCs) and polymorphonuclear MDSCs (PMN-MDSCs) were characterized by flow cytometry (Figure 2A). IR, but not entinostat, increased the abundance of total MDSCs, which was further enhanced by combined treatment (Figure 2B). Entinostat increased the abundance of PMN-MDSCs rather than that of M-MDSCs when combined with IR. The infiltration of PD-L1-expressing MDSCs was significantly higher in the combination treatment group. However, compared to sham treatment, the expression of arginase 1 and TGF-β, which mediate immune suppression, was higher in M-MDSCs but not PMN-MDSCs when co-treated with entinostat and IR (Figure S1).

**Figure 3:**
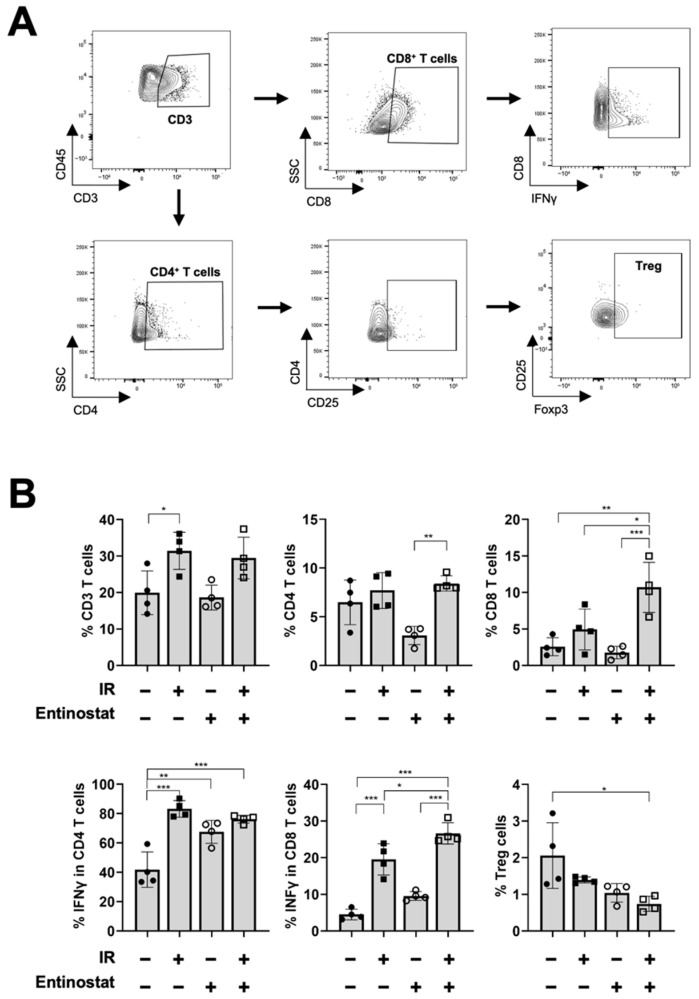
Entinostat increases radiation-induced CD8^+^ T cell infiltration in tumors. (**A**) Gating strategy for the evaluation of the T cell population using flow cytometry. Representative density plots are shown. (**B**) Flow cytometric analysis of T cell populations. Entinostat further enhanced radiation-increased infiltration of IFN-γ-producing CD8^+^ T cells in tumors. The combination of entinostat and IR decreased Treg cells in tumors. Data are mean ± S.D. (*n* ≥ 4). * *p* < 0.05; ** *p* < 0.01; *** *p* < 0.001.

While increasing MDSCs with immunosuppressive activity, entinostat enhanced T-cell-mediated antitumor immunity. We performed flow cytometry to investigate the effect of entinostat on T lymphocytes in tumors harvested 15 d after irradiation (Figure 3A). IR significantly increased the infiltration of CD3^+^ and IFN-γ-producing CD4^+^ and CD8^+^ T cells (Figure 3B). Co-treatment with entinostat further increased IFN-γ-producing CD8+ T cells and decreased the fraction of Tregs (Figure 3B).

### 2.3. Transcriptomic Analysis Reveals That Entinostat Alters Radiation-Mediated Immune Response in the Tumor Microenvironment

**Figure 4:**
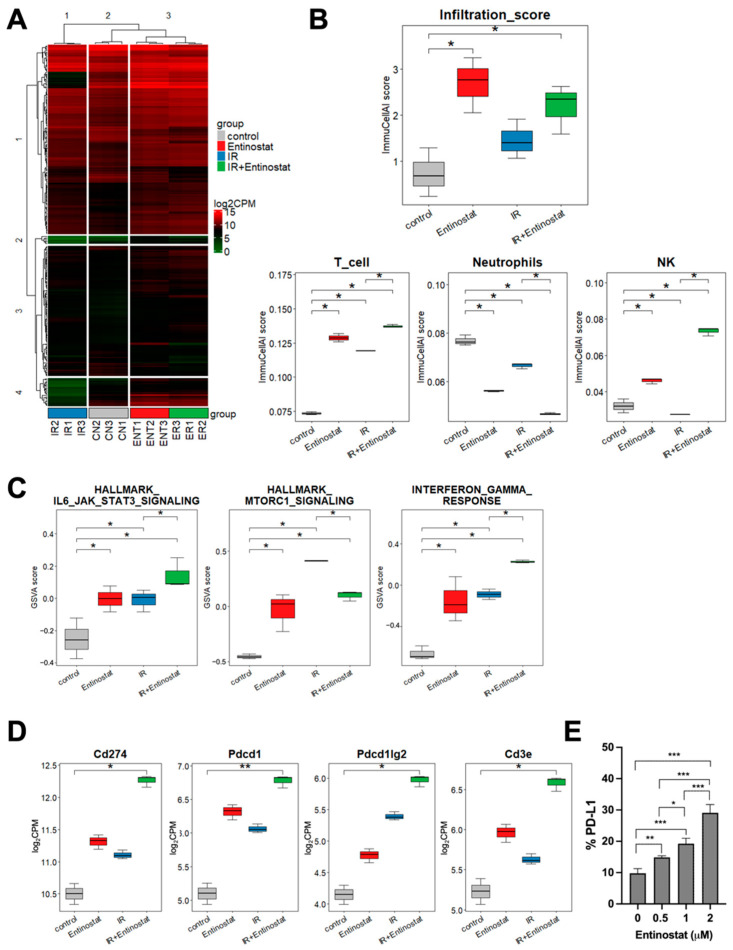
Transcriptomic analysis reveals entinostat-mediated immune modulation in the tumor microenvironment. (**A**) Heatmap representing hierarchical clustering of all DEGs in four groups. (**B**) ImmuCellAI-mouse based estimation of immune cells infiltrating in tumors. (**C**) Comparison of GSVA scores of IL6-JAK-STAT3, mTORC1, and IFN-γ signaling pathways among three groups. (**D**) Comparison of expression levels of PD-1 pathway genes. (**E**) Entinostat induces PD-L1 in LL/2 cells. Dose-dependent induction of PD-L1 in LL/2 cell, which was assessed by flow cytometry. * *p* < 0.05, ** *p* < 0.01, *** *p* < 0.001.

To better understand how entinostat modulates the IR-induced immune response, we performed an RNA-seq analysis of tumors harvested on day 15 of irradiation. Unsupervised hierarchical clustering of 278 differentially expressed genes (DEGs) comparing each treatment group and control showed a closer correlation between the entinostat-treated groups than that between the IR-treated groups (Figure 4A). The ImmuCellAI-mouse analysis estimated that both entinostat and IR treatment increased the infiltration scores relative to the sham control group (Figure 4B). The score of the entinostat and IR combination was higher than that of IR alone but lower than that of entinostat alone. It was estimated that the combined treatment increased the infiltration of T and NK cells and decreased neutrophil infiltration compared to the control and each treatment group (Figure 4B). Figure S2 shows the comparisons of the infiltration scores of the individual immune cell subpopulations. The entinostat group had the highest B cell infiltration, which may contribute to the total infiltration score.

Gene set variation analysis (GSVA) enrichment scores were calculated to discriminate changes in pathway activity among the treatment groups. The group treated with IR had higher GSVA enrichment scores for IL6/JAK/STAT3, mTORC1, and interferon-gamma (IFN-γ) pathways than the control group (Figure 4C). Combined IR and entinostat further increased the GSVA score of IL6/JAK/STAT3 and IFN-γ but decreased that of mTORC1 signaling. Additionally, entinostat modulated various immune-related pathways, as listed in Figure S3. Expression data analysis of each gene showed that the IR-induced PD-1 (CD274), PD-L1 (Pdcd1), and PD-L2 (Pdcd1lg2) expression were further enhanced by the addition of entinostat (Figure 4D). Flow cytometry also confirmed that entinostat increased the population of PD-L1-positive LL/2 cells in vitro in a dose-dependent manner (Figure 4E).

### 2.4. Entinostat Boosts the Combined Effect of Radiation and Anti-PD-1 Immunotherapy

**Figure 5:**
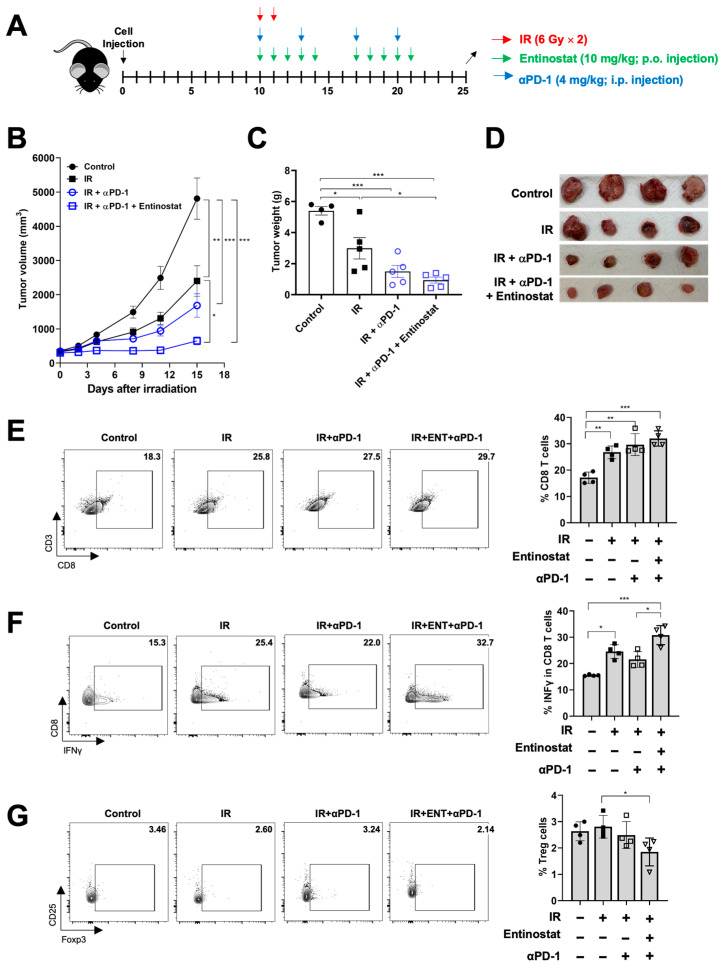
Entinostat sensitizes LL/2 tumors to IR plus anti-PD-1 treatment. (**A**) Scheme for radiation, entinostat, and anti-PD-1 treatments. (**B**) Tumor growth curves showing delayed growth of LL/2 tumors in mice treated with triple combinations. (**C**) Comparison of tumor weight at day 15 after irradiation. (**D**) Photographs of tumors harvested from the mice at day 15 after irradiation. (**E**–**G**) Flow cytometric analysis of CD8^+^ T (**E**), IFN-γ-producing CD8^+^ (**F**), and Treg (**G**) cells infiltrated in tumors. Representative density plots are shown. Data are mean ± S.D. (*n* ≥ 4). * *p* < 0.05; ** *p* < 0.01; *** *p* < 0.001.

Based on the results of the PD-1 signaling induction by the combined treatment, we tested the antitumor effect of a triple combination of entinostat, IR, and anti-PD-1. Anti-PD-1 antibodies were administered twice a week, starting on the same day as irradiation (Figure 5A). As expected, anti-PD-1 augmented radiation-induced tumor growth inhibition (Figure 5B–D), and the addition of entinostat to IR plus anti-PD-1 had a better tumor control effect (Figure 5B–D). Flow cytometry-based immune profiling revealed that anti-PD-1 co-treatment increased CD8^+^ T cell infiltration compared to sham treatment (Figure 5E,F). The triple combination further increased IFN-γ-producing CD8+ T cells in LL/2 tumors, compared to the anti-PD-1/IR combination (Figure 5E,F). In contrast, the fraction of immunosuppressive Treg cells significantly decreased in the triple combination group (Figure 5G).

### 2.5. Potentiation of IR plus Anti-PD1 Combination with Entinostat May Be Caused by Upregulating Type II Interferon Signaling and MHC-II Pathway

**Figure 6:**
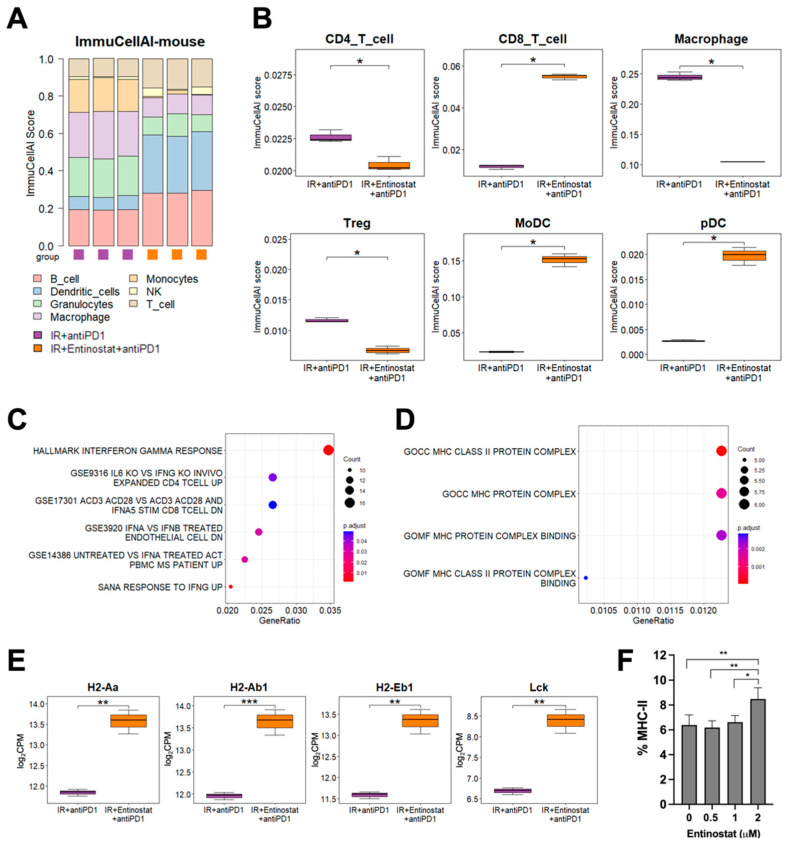
Entinostat potentiates the combination of IR and anti-PD-1 via upregulating IFN-γ and MHC-II pathways (**A**) Comparison of estimated infiltration of different immune cell populations in tumors between IR plus anti-PD-1 group and triple combination group. (**B**) Box plots showing the difference in each immune cell population between the two groups. (**C**) Dot plots showing enrichment of DEGs related to interferon pathways. (**D**) Dot plots showing enrichment of DEGs related to MHC genes between two groups. (**E**) Higher expression levels of MHC-II pathway genes in the triple combination group than those in the IR plus anti-PD-1 group. (**F**) Entinostat induces MHC-II in LL/2 cells. Dose-dependent induction of MHC-II in LL/2 cells, which was assessed by flow cytometry. * *p* < 0.05, ** *p* < 0.01, *** *p* < 0.001.

To understand the mechanism by which entinostat potentiates IR plus anti-PD-1 therapy, we performed RNA-seq analysis of tumors treated with IR or IR/anti-PD-1. Immune cell population prediction showed that adding an entinostat increased the numbers of dendritic cells (DCs) and T cells but decreased granulocytes and macrophages (all *p* < 0.05, Figure 6A). Regarding T cell subsets, the number of CD8^+^ T cells was increased and Treg and CD4^+^ T cells were decreased in the triple combination group compared to the group without entinostat (all *p* < 0.05, Figure 6B). The changes in CD8^+^ T and Treg cells were consistent with the flow cytometry results, as shown in Figure 5. Monocyte-derived DC (MoDCs) and plasmacytoid DC (pDC) were increased by entinostat, although they were not determined by flow cytometry. The ImmunCellAI scores of other cell types, including macrophages and B cells, are presented in Figure S4.

Next, we determined which signaling pathway may be involved in the entinostat-mediated potentiation of IR plus anti-PD-1 therapy. Overrepresentation analysis (ORA) using statistically significant DEGs showed a strong association between IFN-γ signaling-related pathways and entinostat treatment (Figure 6C). A heatmap of DEGs indicated the upregulation of IFN-γ-inducing genes, including CD74 (Figure S5A). Gene ontology analysis and a heatmap of DEGs showed that entinostat increased the expression levels of murine MHC-II genes, such as H2-Aa and H2-Ab1, and Lck, a kinase for T cell activation, in the tumors co-treated with IR/anti-PD-1 (Figure 6D,E and Figure S5B). Flow cytometry analysis also confirmed the direct MHC-II induction by entinostat in the LL/2 cells (Figure 6F).

## 3. Discussion

Cancer immunotherapy based on the PD-1/PD-L1 blockade is a paradigm shift in treating solid cancers. In lung cancer, antibodies targeting PD-1 (nivolumab and pembrolizumab) or PD-L1 (atezolizumab and durvalumab) are used in various clinical settings. RT is a major modality used to destroy cancer cells using high-energy X-rays or particles, and a high-precision RT technique, such as stereotactic body radiation therapy (SBRT), is effective for managing early-stage NSCLC. The success of the PACIFIC trial (NCT02125461) led to concurrent chemoradiation followed by durvalumab as the standard regimen for patients with unresectable stage III NSCLC [2]. Subsequently, various clinical trials evaluating the combination of ICIs and radiotherapy have been conducted, considering the immune-boosting effect of RT. For example, a clinical study (NCT02904954) showed that the neoadjuvant durvalumab with SBRT improved the major pathological response (53.3% vs. 6.7%) in patients with early-stage NSCLC compared to durvalumab monotherapy [24].

Epigenetic drugs, such as HDACis, have been approved for the treatment of hematologic malignancies, including leukemia. Despite many efforts, most epigenetic drugs exhibit promising but limited efficacy against solid cancers. RT has proven to be a good option to combine with HDACi in many solid cancers, including glioblastoma [25], head and neck squamous cell cancer [26], and NSCLC [27]. HDACis downregulate DNA damage response or repair genes, such as ATM, ATR, Ku80, and Rad51, which results in DNA damage accumulation and cell death [28,29]. Entinostat, also known as MS-275, is a benzamide derivative HDAC inhibitor that shows radiosensitizing activity against prostate carcinoma and glioma cells via DNA repair inhibition [30]. Another recent study showed that entinostat increased radiosensitivity by inhibiting DNA damage repair and reactive oxygen species production in PAX3-FOXO1 rhabdomyosarcoma cells in vitro and in vivo [31]. To the best of our knowledge, this is the first study to investigate the combined effects of entinostat and RT on tumor immunology.

The immunomodulatory activity of epigenetic drugs has gained great attention in the cancer immunotherapy era. In the tumor microenvironment, T cell-mediated tumor cell killing is hindered by two major immunosuppressive cells, MDSCs and Tregs, which are therapeutic targets for epigenetic drugs, such as entinostat to enhance anticancer immunotherapy. There are several studies on MDSC modulation of entinostat. Orillion et al. demonstrated that entinostat enhanced the antitumor effect of PD-1 blockade in syngeneic lung and renal cell carcinoma mouse models [19]. Additionally, Christmas et al. showed that the combination of entinostat with ICIs, such as anti-PD-1, significantly empowered T cell-mediated antitumor immunity in HER2^+^ breast cancer and metastatic pancreatic cancer mouse models [21]. In both studies, the entinostat/anti-PD-1 combination increased infiltration of CD8^+^ effector T cells and MDSCs in the TME but diminished MDSC’s immunosuppressive activity, compared to the control. Our flow cytometry data showed that entinostat enhanced the antitumor effect of IR by increasing the number of cytotoxic CD8^+^ T cells in tumors. The entinostat/IR combination also increased total MDSC levels, but the PMN-to-M-MDSC ratio also increased. It was recently reported that a shift in the balance between M-MDSCs and PMN-MDSCs is one of the mechanisms of entinostat-induced immune activation [13,32]. Another study showed that entinostat selectively inhibited PMN-MDSCs but not M-MDSCs [20]. Unlike other studies, we did not observe that entinostat affected Arg1 and TGF-β expressions in MDSCs. Therefore, regulation of MDSCs by entinostat is context-dependent, and IR-mediated recruitment or activity of MDSCs might not be directly related to entinostat-induced antitumor immunity. It is necessary to test whether the combination of entinostat and radiation modulates the production of reactive oxygen and nitrogen species in MDSCs in terms of MDSC activity for T-cell suppression.

Foxp3^+^ Tregs are a major immune cell type with strong immunosuppressive activity. Treg suppression by entinostat has been reported in prostate cancer models as well as in healthy donors [33,34]. However, two recent studies using a combination of entinostat and anti-PD-1 have demonstrated that the inhibitory effect of entinostat on Tregs is too modest to explain the enhanced antitumor activity [19,21]. A recent study using bladder cancer models supports the notion that entinostat negatively modulates both Tregs and MDSCs [13]. In the LL/2 model, we found that the entinostat/IR combination decreased Treg levels in the TME compared to the control, and adding entinostat to anti-PD-1/IR decreased Tregs compared to IR alone. These data suggest that entinostat may play a role in antitumor immune activation in the TME by inhibiting Tregs.

Genome-wide gene expression analysis is a valuable tool for identifying the molecular mechanisms underlying the therapeutic effects against cancer. We used a tool called ImmuCellAI, which is designed to identify 24 immune cell subsets based on a gene expression dataset from RNA-seq data, and found similar data to flow cytometry-based immune profiling data: increased CD8^+^ T cells and decreased Tregs in the triple combination group. The GSVA score for the molecular signature database revealed significant enrichment of IL6/JAK/STAT signaling in the IR/entinostat group, which may be related to IFN-γ induction in the corresponding group. IR-increased GSVA score of mTORC1 signaling was suppressed by adding entinostat, which may be supported by previous reports that entinostat suppresses cancer cell growth via PI3K/AKT/mTOR signaling inhibition [31,35]. However, the effects of mTOR inhibition on immune cells remain unclear. Entinostat-induced PD-L1 expression was observed in bladder and ovarian cancers [13,36], which was consistent with our data showing that entinostat significantly upregulated the PD-1/PD-L1 pathway genes. This explains why the triple combination of entinostat/IR/PD-1 blockade was the best approach for tumor control in our experimental settings. Our data showed significant upregulation of MHC-II genes in the triple combination group compared to that in the IR/anti-PD-1 group. It was previously shown that entinostat promotes the expression of MHC-II genes through the induction of Ciita, a master regulator, in ovarian cancer models [36,37]. MHC-II induction by entinostat in LL/2 cells was confirmed by flow cytometry. Besides professional antigen-presenting cells, including DCs, MHC-II induction in cancer cells sensitizes tumors to anti-PD-1 therapy in lung adenocarcinomas [38]. Based on our data showing an increase in MHC-II expression and DCs in the triple combination group, it is likely that the upregulation of MHC-II genes by entinostat may enhance IR/PD-1 therapy.

Our study has several limitations. Our hypothesis was tested using a syngeneic mouse model. Further studies are needed to determine to know whether triple combination therapy with entinostat/IR/anti-PD-1 can be applied to different tumor model systems. Additionally, the optimal conditions for the synergism of the triple combinations need to be investigated. Biochemical assays are also required to validate the pathways derived from bioinformatic transcriptomic data (e.g., immunohistochemistry or western blot for STAT3 phosphorylation). A recent comprehensive study using single-cell RNA sequencing revealed that entinostat modulates MDSCs and tumor-associated macrophages, shifting the balance toward favorable conditions for ICIs [32]. Thus, the single-cell RNA sequencing approach for specialized immune subtypes in the TME may provide deep insight into how entinostat enhances the antitumor efficacy of the IR/anti-PD-1 combination and can offer clues for developing new strategies for clinical trials.

## 4. Materials and Methods

### 4.1. Cell Culture

Murine Lewis lung carcinoma (LL/2-luc) cells were purchased from Perkin Elmer (Waltham, MA, USA) and cultured as previously described [23].

### 4.2. Animal Models

All animal experiments were reviewed and approved by the Institutional Animal Care and Use Committee (IACUC) of the Samsung Biomedical Research Institute (SBRI) (ID:20180625002; approval date: 3 July 2018 and ID:20190926001; approval date: 18 October 2019). Four-week-old male C57BL/6 mice were obtained from Orient Bio, Inc. (Gapyeong, Gyeonggi, Republic of Korea). The animal study was performed in compliance with the ARRIVE guidelines [39].

For LL/2 tumor models, 1 × 106 LL/2 cells were subcutaneously injected into the right hind legs of C57BL/6 mice. The mice were randomized on day 7 after tumor injection. Tumor-bearing legs were irradiated with a fraction dose of 6 Gy X-rays for two consecutive days (total dose of 12 Gy). Mice were orally administered entinostat (Selleck Chemicals, Houston, TX, USA) at 10 mg/kg daily for 2 weeks and/or intraperitoneally administered anti-PD-1 antibody (Bio X Cell, West Lebanon, NH, USA) at 4 mg/kg twice a week for 2 weeks. Tumor volumes were measured twice a week using a digital caliper and were calculated as follows: volume = (width^2^ × length)/2.

### 4.3. Irradiation

For tumor irradiation, 6 MV photon beams were delivered at a dose rate of 3.96 Gy/min using a Varian Clinac 6EX linear accelerator (Varian Medical System, Palo Alto, CA, USA). The mice were anesthetized with intraperitoneal injections of 50 mg/kg zolazepam/tiletamine and 10 mg/kg xylazine immediately before irradiation. To avoid unnecessary radiation exposure to other organs, only tumor-implanted hind legs were placed inside the radiation field (32 cm × 7 cm), which was achieved by collimating the jaws.

### 4.4. Flow Cytometry

Harvested tumors were cut into small pieces and dissociated using a Tumor Dissociation Kit according to the manufacturer’s instructions (Miltenyi Biotec, Auburn, CA, USA). Red blood cells were lysed using BD Pharm LyseTM lysing buffer (BD Bioscience, San Jose, CA, USA). Cell suspensions were stained with fluorescence-conjugated antibodies specific for CD45, CD11b, Ly6G, Ly6C, CD3, CD4, CD8, CD25 (BD Biosciences), and PD-L1 (eBioscience, San Diego, CA, USA). For intracellular staining, cells were fixed and permeabilized with fixation/permeabilization buffer (eBioscience) and stained with antibodies specific for Arg1, TGF-β, IFN-γ, and Foxp3 (BD Bioscience). Flow cytometric analysis was performed using a BD FACSVerse flow cytometer (BD Bioscience) and FlowJo software version 10.6.1 (BD Bioscience).

### 4.5. RNA Sequence Analysis

Whole transcriptome sequencing was performed on fresh mouse tissues. RNA was extracted using Qiagen RNeasy Mini Kit. Sequencing libraries were prepared using the TruSeq RNA Library Prep Kit v2 (Illumina, Inc., San Diego, CA, USA) following the manufacturer’s protocols. Paired-end sequencing of RNA libraries was performed using the HiSeq 2500 sequencing platform (Illumina, Inc., San Diego, CA, USA). After trimming poor-quality bases from the FASTQ files, we aligned the reads to the mouse reference genome (mm10) using STAR (v2.5.2b) [40] and estimated gene expression using RSEM (v1.3) [41]. Comparisons of the control versus treatment groups or between treatment groups were performed using the edgeR R package (v3.36.0) [42]. Counts extracted from the RSEM outputs were normalized using the TMM method after excluding low-expressed counts, and generalized linear models were used to identify DEGs. In all cases, DEGs were defined as those with unadjusted *p*-values under 0.05, absolute log fold change over 1, and average log_2_-counts per million (log_2_CPM) over 2. Gene set enrichment was analyzed using the mouse msigdbr R package (v7.4.1) in three ways. ORA and gene set enrichment analysis was performed using the clusterProfiler R package (v4.2.0) [43] with false discovery rate (FDR)-corrected *p*-values under 0.05. The Gene Set Variation (GSVA) score of the gene sets was calculated using the GSVA R package (v1.34.0) [44]. Differentially expressed gene sets using the GSVA score were defined as those with unadjusted *p*-values under 0.05, and absolute log fold change over 1. Mouse immune cell abundance was predicted using ImmuCellAI-mouse (http://bioinfo.life.hust.edu.cn/ImmuCellAI-mouse/#!/, accessed on 16 March 2022) [45]. All statistical analyses and plotting of the RNA sequencing data were performed using R version 4.1.2.

### 4.6. Statistics

Statistical analyses were performed using GraphPad Prism 9.4.1 (GraphPad Software, San Diego, CA, USA). Statistical differences between the groups were determined using a one-way analysis of variance with Bonferroni’s comparison test. Statistical significance was set at *p* < 0.05.

## 5. Conclusions

In this study, we showed that the histone deacetylase inhibitor entinostat potentiated the efficacy of radiotherapy and anti-PD-1 using a murine syngeneic LL/2 tumor model. Through immune profiling and transcriptomic analysis, we found that entinostat might facilitate IFN-γ signaling and MHC-II gene expression, thereby improving the efficacy of IR plus anti-PD-1 therapy. Thus, adding an entinostat to the combination of IR and ICIs may be a promising strategy to fully activate the antitumor immune response and optimize efficacy.

## Data Availability

Not applicable.

## Supplementary Materials

The following supporting information can be downloaded at: https://www.mdpi.com/article/10.3390/ijms232415539/s1.

## References

[1] Zhang Z., Liu X., Chen D., Yu J. (2022). Radiotherapy combined with immunotherapy: The dawn of cancer treatment. Signal Transduct. Target. Ther..

[2] Antonia S.J., Villegas A., Daniel D., Vicente D., Murakami S., Hui R., Yokoi T., Chiappori A., Lee K.H., De Wit M. (2017). Durvalumab after Chemoradiotherapy in Stage III Non-Small-Cell Lung Cancer. N. Engl. J. Med..

[3] Spigel D.R., Faivre-Finn C., Gray J.E., Vicente D., Planchard D., Paz-Ares L., Vansteenkiste J.F., Garassino M.C., Hui R., Quantin X. (2022). Five-Year Survival Outcomes From the PACIFIC Trial: Durvalumab After Chemoradiotherapy in Stage III Non–Small-Cell Lung Cancer. J. Clin. Oncol..

[4] Bao L., Diao H., Dong N., Su X., Wang B., Mo Q., Yu H., Wang X., Chen C. (2016). Histone deacetylase inhibitor induces cell apoptosis and cycle arrest in lung cancer cells via mitochondrial injury and p53 up-acetylation. Cell Biol. Toxicol..

[5] Tang Y.-A., Wen W.-L., Chang J.-W., Wei T.-T., Tan Y.-H.C., Salunke S., Chen C.-T., Chen C.-S., Wang Y.-C. (2010). A Novel Histone Deacetylase Inhibitor Exhibits Antitumor Activity via Apoptosis Induction, F-Actin Disruption and Gene Acetylation in Lung Cancer. PLoS ONE.

[6] Ramalingam S.S., Maitland M.L., Frankel P., Argiris A.E., Koczywas M., Gitlitz B., Thomas S., Espinoza-Delgado I., Vokes E.E., Gandara D.R. (2010). Carboplatin and Paclitaxel in Combination With Either Vorinostat or Placebo for First-Line Therapy of Advanced Non–Small-Cell Lung Cancer. J. Clin. Oncol..

[7] Reguart N., Rosell R., Cardenal F., Cardona A.F., Isla D., Palmero R., Moran T., Rolfo C., Pallarès M.C., Insa A. (2014). Phase I/II trial of vorinostat (SAHA) and erlotinib for non-small cell lung cancer (NSCLC) patients with epidermal growth factor receptor (EGFR) mutations after erlotinib progression. Lung Cancer.

[8] Munshi A., Tanaka T., Hobbs M.L., Tucker S.L., Richon V.M., Meyn R.E. (2006). Vorinostat, a histone deacetylase inhibitor, enhances the response of human tumor cells to ionizing radiation through prolongation of γ-H2AX foci. Mol. Cancer Ther..

[9] Rivera S., Leteur C., Mégnin F., Law F., Martins I., Kloos I., Depil S., Modjtahedi N., Perfettini J.L., Hennequin C. (2017). Time dependent modulation of tumor radiosensitivity by a pan HDAC inhibitor: Abexinostat. Oncotarget.

[10] Choi C., Lee G., Son A., Yoo G., Yu J., Park H. (2021). Downregulation of Mcl-1 by Panobinostat Potentiates Proton Beam Therapy in Hepatocellular Carcinoma Cells. Cells.

[11] Yu J.I., Choi C., Shin S.-W., Son A., Lee G.-H., Kim S.-Y., Park H.C. (2017). Valproic Acid Sensitizes Hepatocellular Carcinoma Cells to Proton Therapy by Suppressing NRF2 Activation. Sci. Rep..

[12] Ritter C., Fan K., Paschen A., Hadrup S.R., Ferrone S., Nghiem P., Ugurel S., Schrama D., Becker J.C. (2017). Epigenetic priming restores the HLA class-I antigen processing machinery expression in Merkel cell carcinoma. Sci. Rep..

[13] Truong A.S., Zhou M., Krishnan B., Utsumi T., Manocha U., Stewart K.G., Beck W., Rose T.L., Milowsky M.I., He X. (2021). Entinostat induces antitumor immune responses through immune editing of tumor neoantigens. J. Clin. Investig..

[14] Briere D., Sudhakar N., Woods D.M., Hallin J., Engstrom L.D., Aranda R., Chiang H., Sodré A.L., Olson P., Weber J.S. (2018). The class I/IV HDAC inhibitor mocetinostat increases tumor antigen presentation, decreases immune suppressive cell types and augments checkpoint inhibitor therapy. Cancer Immunol. Immunother..

[15] Woods D.M., Sodré A.L., Villagra A., Sarnaik A.A., Sotomayor E.M., Weber J. (2015). HDAC Inhibition Upregulates PD-1 Ligands in Melanoma and Augments Immunotherapy with PD-1 Blockade. Cancer Immunol. Res..

[16] Li X., Su X., Liu R., Pan Y., Fang J., Cao L., Feng C., Shang Q., Chen Y., Shao C. (2021). HDAC inhibition potentiates anti-tumor activity of macrophages and enhances anti-PD-L1-mediated tumor suppression. Oncogene.

[17] Booth L., Roberts J.L., Poklepovic A., Kirkwood J., Dent P. (2017). HDAC inhibitors enhance the immunotherapy response of melanoma cells. Oncotarget.

[18] Wang H.-F., Ning F., Liu Z.-C., Wu L., Li Z.-Q., Qi Y.-F., Zhang G., Wang H.-S., Cai S.-H., Du J. (2017). Histone deacetylase inhibitors deplete myeloid-derived suppressor cells induced by 4T1 mammary tumors in vivo and in vitro. Cancer Immunol. Immunother..

[19] Orillion A., Hashimoto A., Damayanti N., Shen L., Adelaiye-Ogala R., Arisa S., Chintala S., Ordentlich P., Kao C., Elzey B. (2017). Entinostat Neutralizes Myeloid-Derived Suppressor Cells and Enhances the Antitumor Effect of PD-1 Inhibition in Murine Models of Lung and Renal Cell Carcinoma. Clin. Cancer Res..

[20] Hashimoto A., Fukumoto T., Zhang R., Gabrilovich D. (2020). Selective targeting of different populations of myeloid-derived suppressor cells by histone deacetylase inhibitors. Cancer Immunol. Immunother..

[21] Christmas B.J., Rafie C.I., Hopkins A.C., Scott B.A., Ma H.S., Cruz K.A., Woolman S., Armstrong T.D., Connolly R.M., Azad N.A. (2018). Entinostat Converts Immune-Resistant Breast and Pancreatic Cancers into Checkpoint-Responsive Tumors by Reprogramming Tumor-Infiltrating MDSCs. Cancer Immunol. Res..

[22] Borcoman E., Kamal M., Marret G., Dupain C., Castel-Ajgal Z., Le Tourneau C. (2021). HDAC Inhibition to Prime Immune Checkpoint Inhibitors. Cancers.

[23] Kim Y., Choi C., Park J.H., Ahn W.-G., Shin S.-W., Kim S.-Y., Noh J.M. (2022). Immunomodulatory effect of splenectomy in lung cancer mouse xenograft models receiving radiation therapy. Radiat. Oncol. J..

[24] Altorki N.K., McGraw T.E., Borczuk A.C., Saxena A., Port J.L., Stiles B.M., Lee B.E., Sanfilippo N.J., Scheff R.J., Pua B.B. (2021). Neoadjuvant durvalumab with or without stereotactic body radiotherapy in patients with early-stage non-small-cell lung cancer: A single-centre, randomised phase 2 trial. Lancet Oncol..

[25] Kim J.H., Shin J.H., Kim I.H. (2004). Susceptibility and radiosensitization of human glioblastoma cells to trichostatin A, a histone deacetylase inhibitor. Int. J. Radiat. Oncol..

[26] Zhang Y., Jung M., Dritschilo A., Jung M. (2004). Enhancement of Radiation Sensitivity of Human Squamous Carcinoma Cells by Histone Deacetylase Inhibitors. Radiat. Res..

[27] Geng L., Cuneo K.C., Fu A., Tu T., Atadja P.W., Hallahan D.E. (2006). Histone Deacetylase (HDAC) Inhibitor LBH589 Increases Duration of γ-H2AX Foci and Confines HDAC4 to the Cytoplasm in Irradiated Non–Small Cell Lung Cancer. Cancer Res..

[28] Shirbhate E., Patel P., Patel V.K., Veerasamy R., Sharma P.C., Rajak H. (2020). The combination of histone deacetylase inhibitors and radiotherapy: A promising novel approach for cancer treatment. Futur. Oncol..

[29] Groselj B., Sharma N.L., Hamdy F.C., Kerr M., Kiltie A.E. (2013). Histone deacetylase inhibitors as radiosensitisers: Effects on DNA damage signalling and repair. Br. J. Cancer.

[30] Camphausen K., Burgan W., Cerra M., Oswald K.A., Trepel J.B., Lee M.-J., Tofilon P.J. (2004). Enhanced Radiation-Induced Cell Killing and Prolongation of γH2AX Foci Expression by the Histone Deacetylase Inhibitor MS-275. Cancer Res..

[31] Cassandri M., Pomella S., Rossetti A., Petragnano F., Milazzo L., Vulcano F., Camero S., Codenotti S., Cicchetti F., Maggio R. (2021). MS-275 (Entinostat) Promotes Radio-Sensitivity in PAX3-FOXO1 Rhabdomyosarcoma Cells. Int. J. Mol. Sci..

[32] Sidiropoulos D.N., Rafie C.I., Jang J.K., Castanon S., Baugh A.G., Gonzalez E., Christmas B.J., Narumi V.H., Davis-Marcisak E.F., Sharma G. (2022). Entinostat Decreases Immune Suppression to Promote Antitumor Responses in a HER2^+^ Breast Tumor Microenvironment. Cancer Immunol. Res..

[33] Shen L., Ciesielski M., Ramakrishnan S., Miles K.M., Ellis L., Sotomayor P., Shrikant P., Fenstermaker R., Pili R. (2012). Class I Histone Deacetylase Inhibitor Entinostat Suppresses Regulatory T Cells and Enhances Immunotherapies in Renal and Prostate Cancer Models. PLoS ONE.

[34] Akimova T., Ge G., Golovina T., Mikheeva T., Wang L., Riley J.L., Hancock W.W. (2010). Histone/protein deacetylase inhibitors increase suppressive functions of human FOXP3+ Tregs. Clin. Immunol..

[35] Ma S., Liu T., Xu L., Wang Y., Zhou J., Huang T., Li P., Liu H., Zhang Y., Zhou X. (2019). Histone deacetylases inhibitor MS-275 suppresses human esophageal squamous cell carcinoma cell growth and progression via the PI3K/Akt/mTOR pathway. J. Cell. Physiol..

[36] Smith H.J., McCaw T.R., Londono A.I., Katre A.A., Meza-Perez S., Yang E.S., Forero A., Buchsbaum N.J., Randall T.D., Straughn J.J.M. (2018). The antitumor effects of entinostat in ovarian cancer require adaptive immunity. Cancer.

[37] Turner T.B., Meza-Perez S., Londoño A., Katre A., Peabody J.E., Smith H.J., Forero A., Norian L.A., Michael Straughn J., Buchsbaum D.J. (2017). Epigenetic modifiers upregulate MHC II and impede ovarian cancer tumor growth. Oncotarget.

[38] Johnson A.M., Bullock B., Neuwelt A.J., Poczobutt J.M., Kaspar R.E., Li H.Y., Kwak J.W., Hopp K., Weiser-Evans M.C.M., Heasley L.E. (2020). Cancer Cell–Intrinsic Expression of MHC Class II Regulates the Immune Microenvironment and Response to Anti–PD-1 Therapy in Lung Adenocarcinoma. J. Immunol..

[39] du Sert N.P., Ahluwalia A., Alam S., Avey M.T., Baker M., Browne W.J., Clark A., Cuthill I.C., Dirnagl U., Emerson M. (2020). Reporting animal research: Explanation and elaboration for the ARRIVE guidelines 2.0. PLOS Biol..

[40] Dobin A., Davis C.A., Schlesinger F., Drenkow J., Zaleski C., Jha S., Batut P., Chaisson M., Gingeras T.R. (2013). STAR: Ultrafast universal RNA-seq aligner. Bioinformatics.

[41] Li B., Dewey C.N. (2011). RSEM: Accurate transcript quantification from RNA-Seq data with or without a reference genome. BMC Bioinform..

[42] Robinson M.D., McCarthy D.J., Smyth G.K. (2010). EdgeR: A Bioconductor package for differential expression analysis of digital gene expression data. Bioinformatics.

[43] Wu T., Hu E., Xu S., Chen M., Guo P., Dai Z., Feng T., Zhou L., Tang W., Zhan L. (2021). clusterProfiler 4.0: A universal enrichment tool for interpreting omics data. Innovation.

[44] Hänzelmann S., Castelo R., Guinney J. (2013). GSVA: Gene set variation analysis for microarray and RNA-Seq data. BMC Bioinform..

[45] Miao Y.-R., Xia M., Luo M., Luo T., Yang M., Guo A.-Y. (2021). ImmuCellAI-mouse: A tool for comprehensive prediction of mouse immune cell abundance and immune microenvironment depiction. Bioinformatics.

