# Supplementary material for: Immunomodulation of HDAC Inhibitor Entinostat Potentiates the Anticancer Effects of Radiation and PD-1 Blockade in the Murine Lewis Lung Carcinoma Model
Source: Int J Mol Sci. 2022 Dec 8;23(24):15539. doi: 10.3390/ijms232415539 (PMC9779092; doi:10.3390/ijms232415539)
Supplement: Supplementary file 1 [file ijms-23-15539-s001.zip › ijms-2013547-supplementary.pdf]

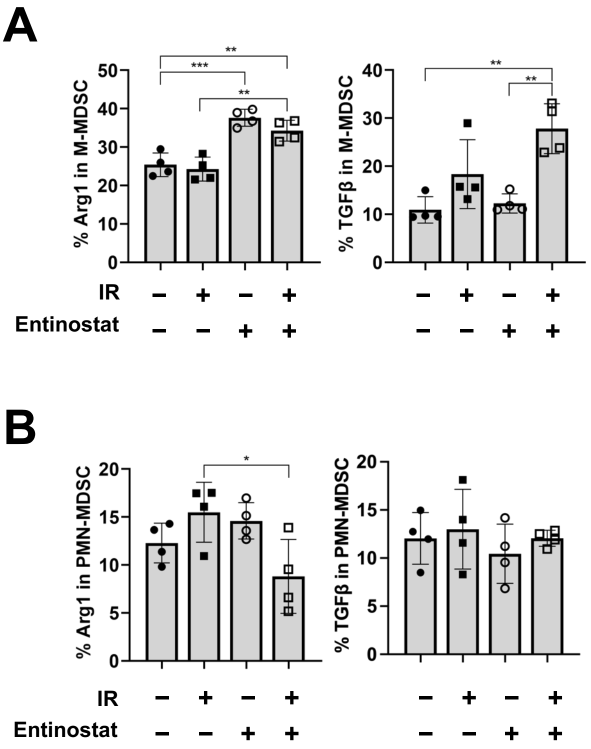

Figure S1. Comparison of arginase 1 and TGFβ expressions in MDSCs isolated from tumors treated with entinostat and/or IR. (A) M-MDSC. **(B)** PMN-MDSC.

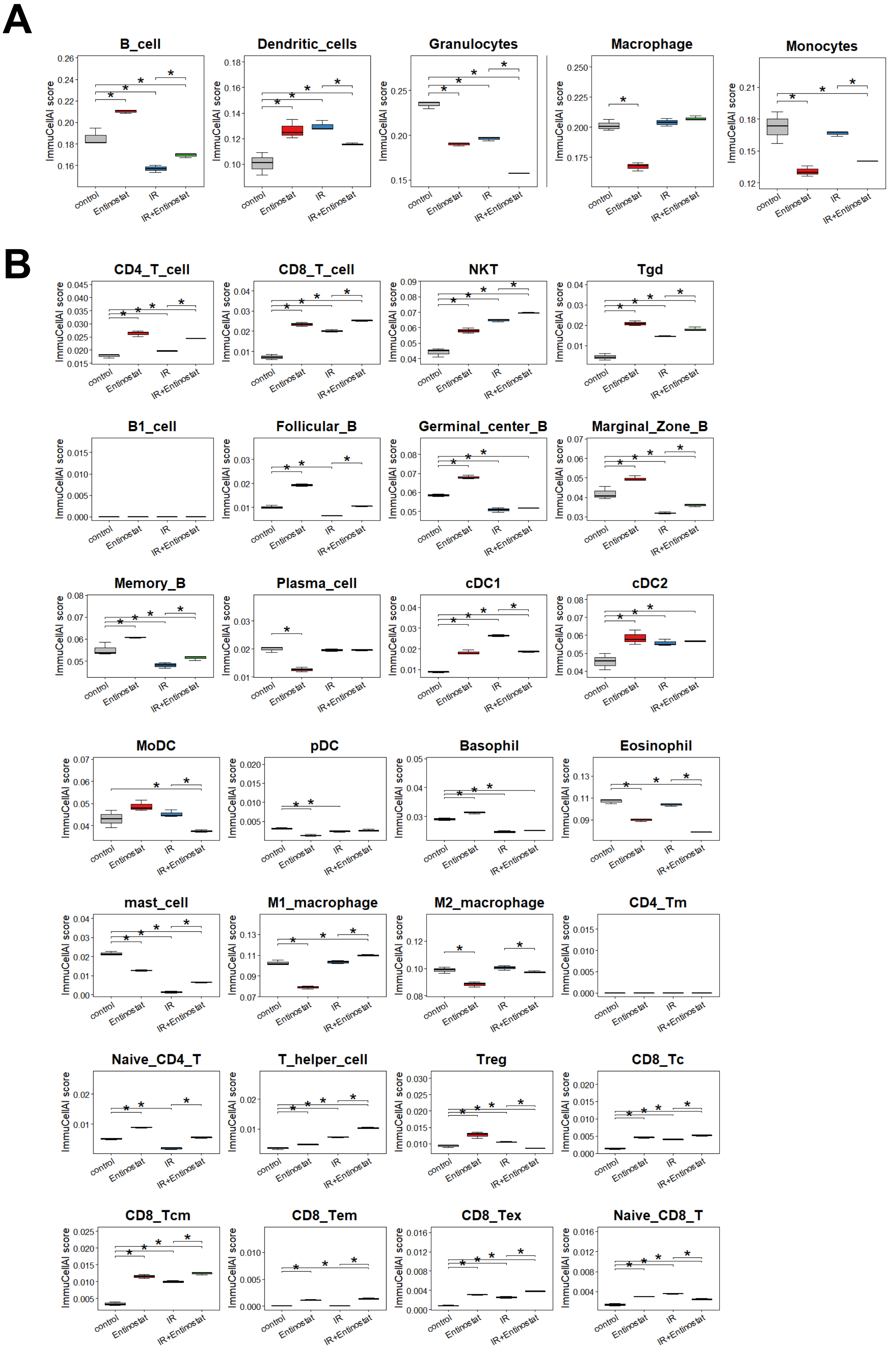

Figure S2. Comparison of estimated infiltration of different immune cell populations in tumors mediated by entinostat. (A) Box plots showing difference in seven major immune cell types between the four groups. **(B)** Box plots showing difference in immune cell subtypes between the four groups. * $p < 0.05$

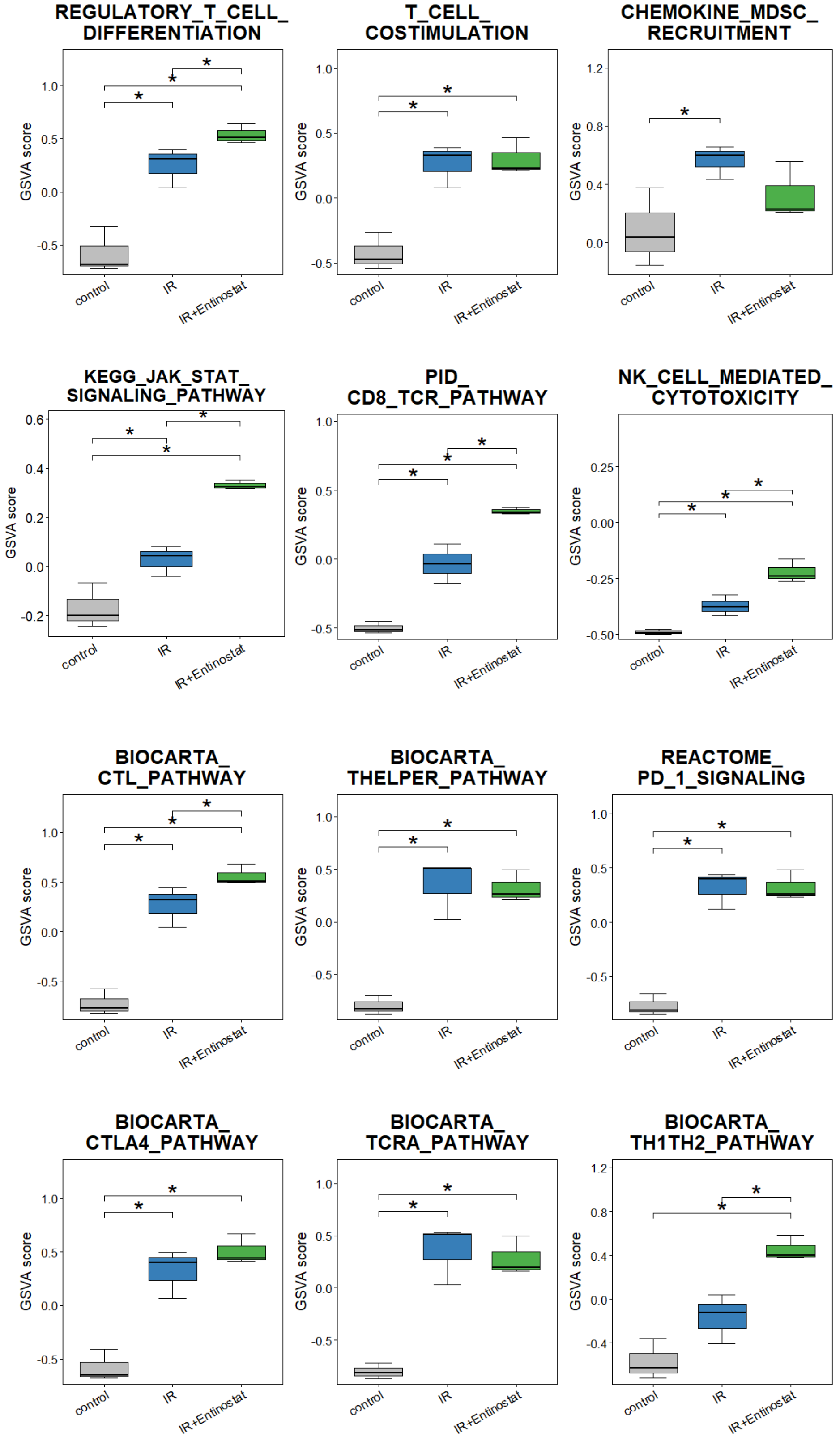

Figure S3. Comparison of GSVA scores in T cell-related pathway and MDSC pathway among three groups. * $p < 0.05$.

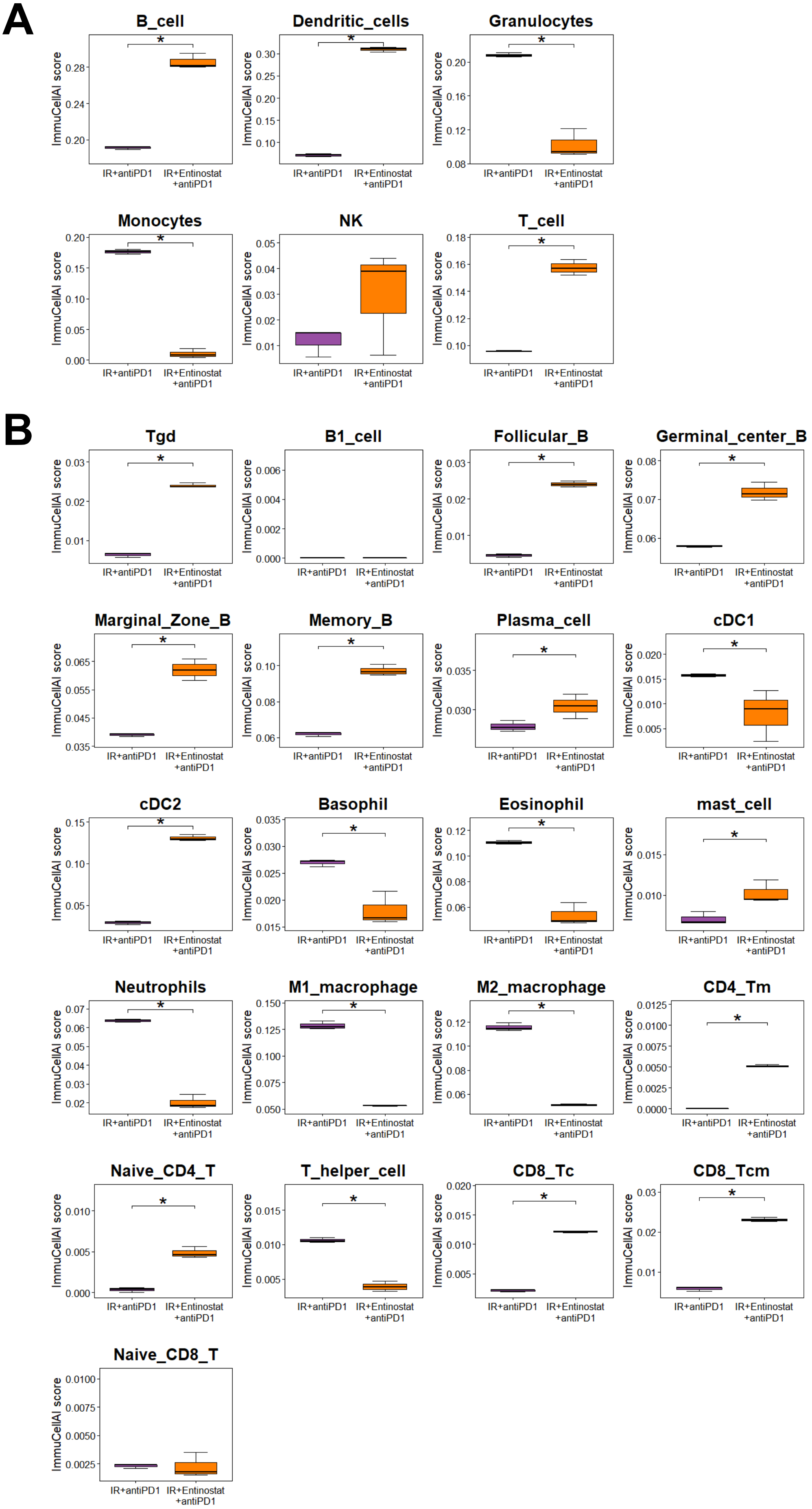

Figure S4. Comparison of estimated infiltration of different immune cell populations in tumors between IR plus anti-PD-1 group and triple combination group. (A) Box plots showing difference in seven major immune cell types between the two groups. **(B)** Box plots showing difference in immune cell subtypes between the two groups. * $p < 0.05$

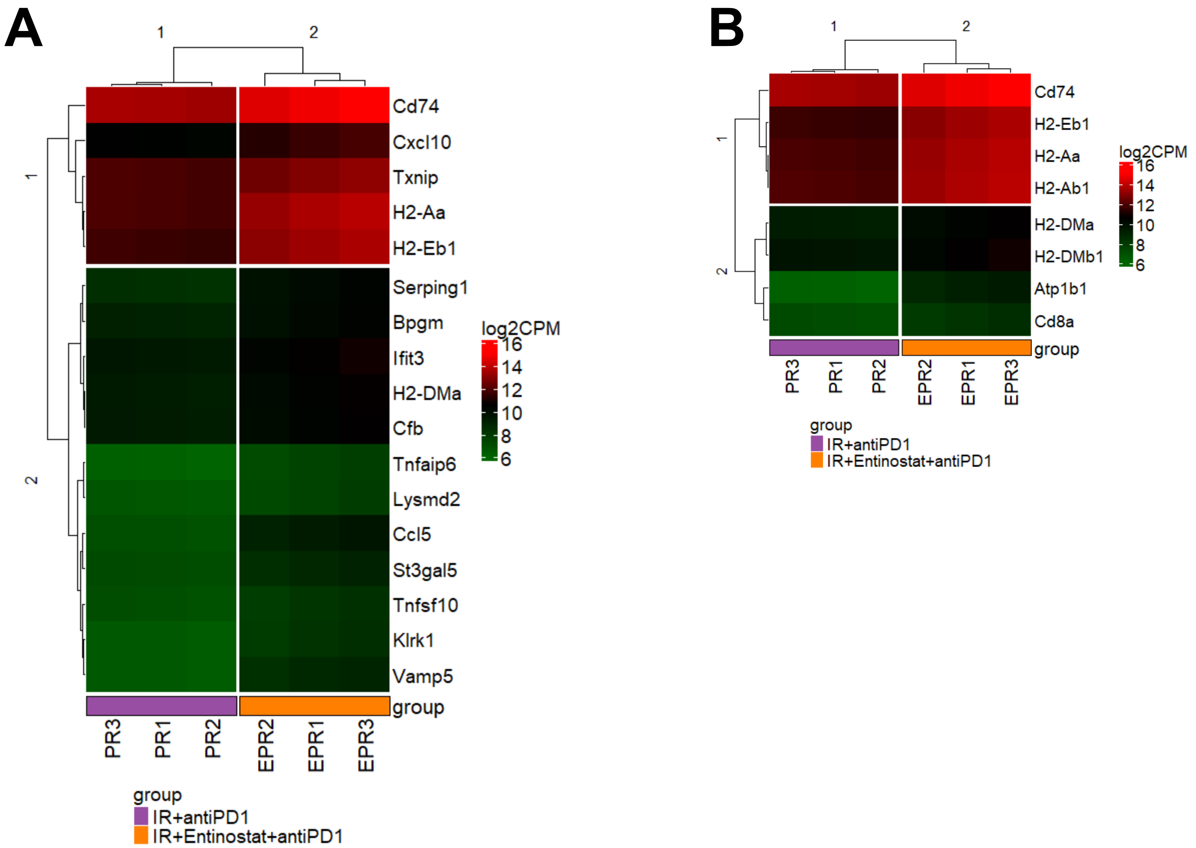

Figure S5. Heatmap with DEGs between IR plus anti-PD-1 group and triple combination group. **(A)** IFNγ-related gene sets. **(B)** MHC-II gene sets.
